# Supplementary material for: Blood meals from ‘dead-end’ vertebrate hosts enhance transmission potential of malaria-infected mosquitoes
Source: One Health. 2023 Jun 9;17:100582. doi: 10.1016/j.onehlt.2023.100582 (PMC10665158; doi:10.1016/j.onehlt.2023.100582)
Supplement: Supplementary Fig. 1 — Overview of study design (for detailed description, refer to ‘Study design’ section under ‘Methods’). [file mmc1.pdf]

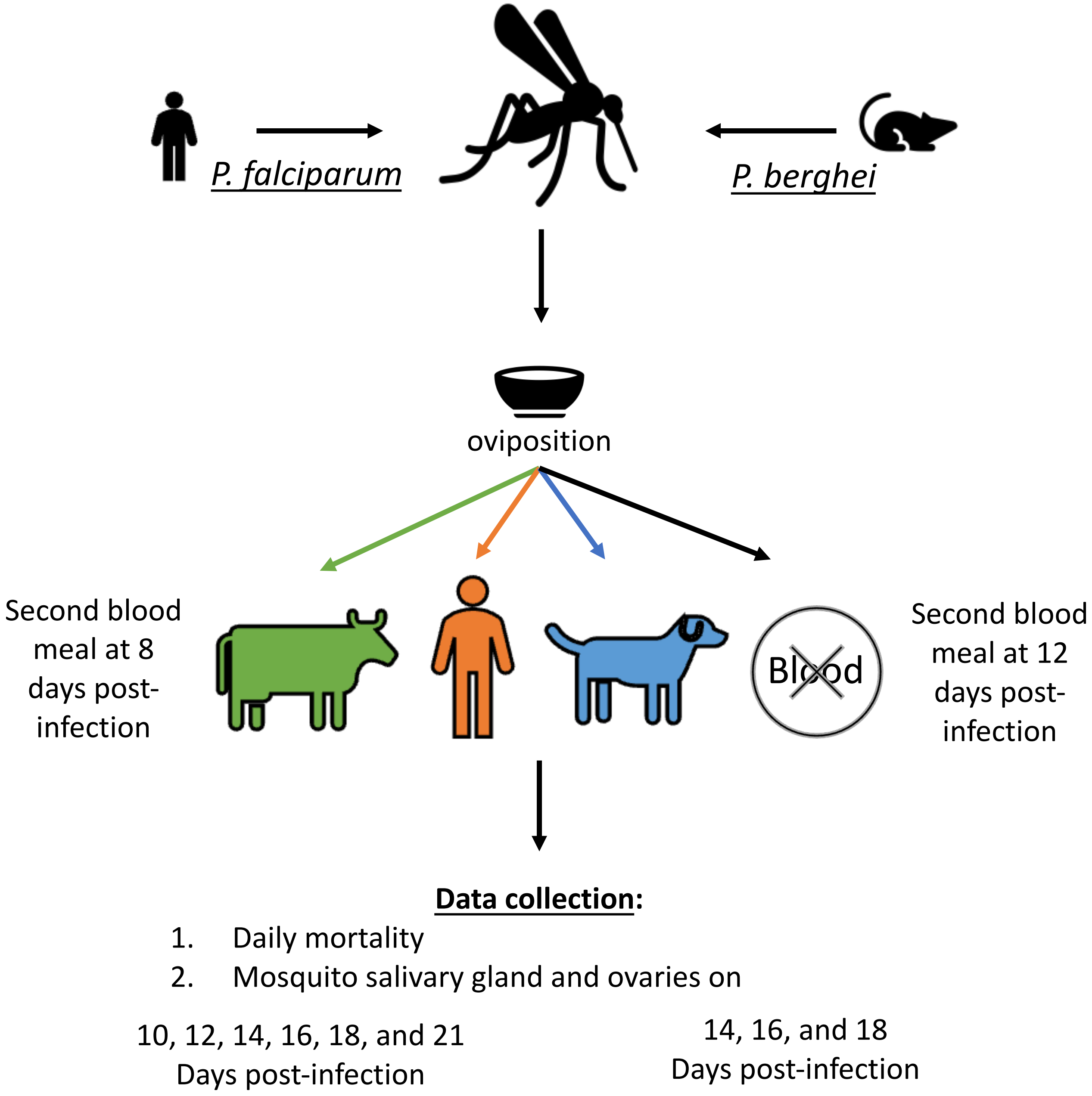
P. falciparum
P. berghei
oviposition
Second blood meal at 8 days post-infection
Blood
Second blood meal at 12 days post-infection
Data collection:
1. Daily mortality
2. Mosquito salivary gland and ovaries on
10, 12, 14, 16, 18, and 21 Days post-infection
14, 16, and 18 Days post-infection
