# Supplementary figures and images for: Blood meals from ‘dead-end’ vertebrate hosts enhance transmission potential of malaria-infected mosquitoes
Source: One Health. 2023 Jun 9;17:100582. doi: 10.1016/j.onehlt.2023.100582 (PMC10665158; doi:10.1016/j.onehlt.2023.100582)

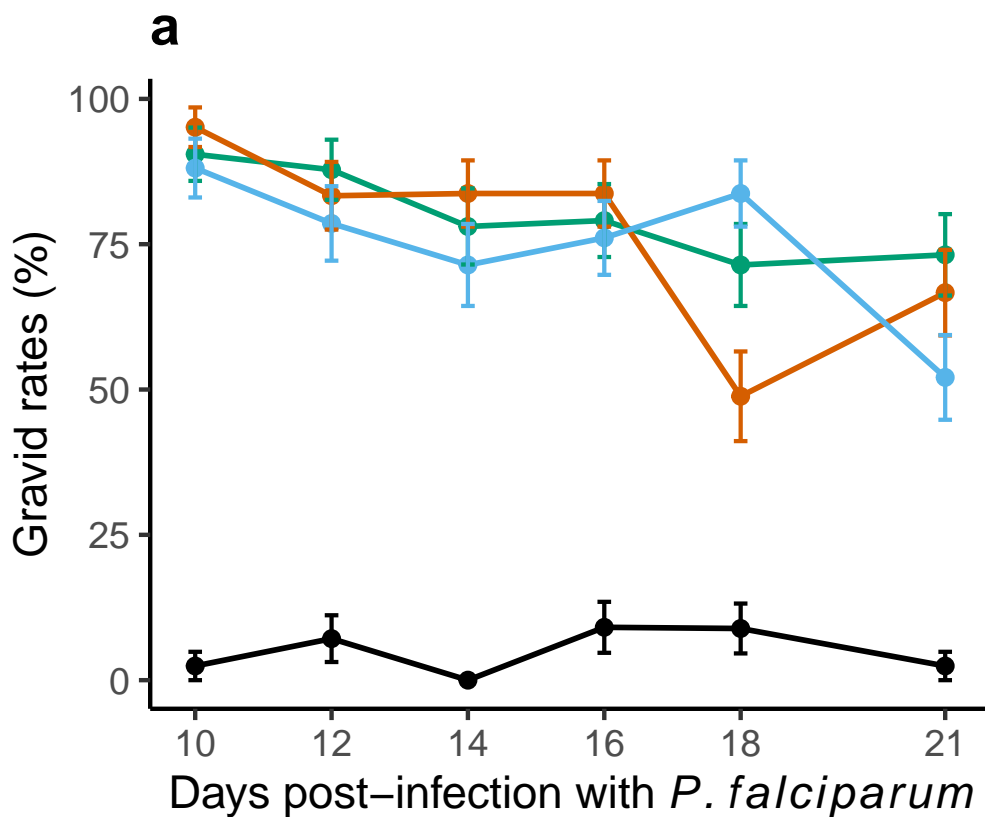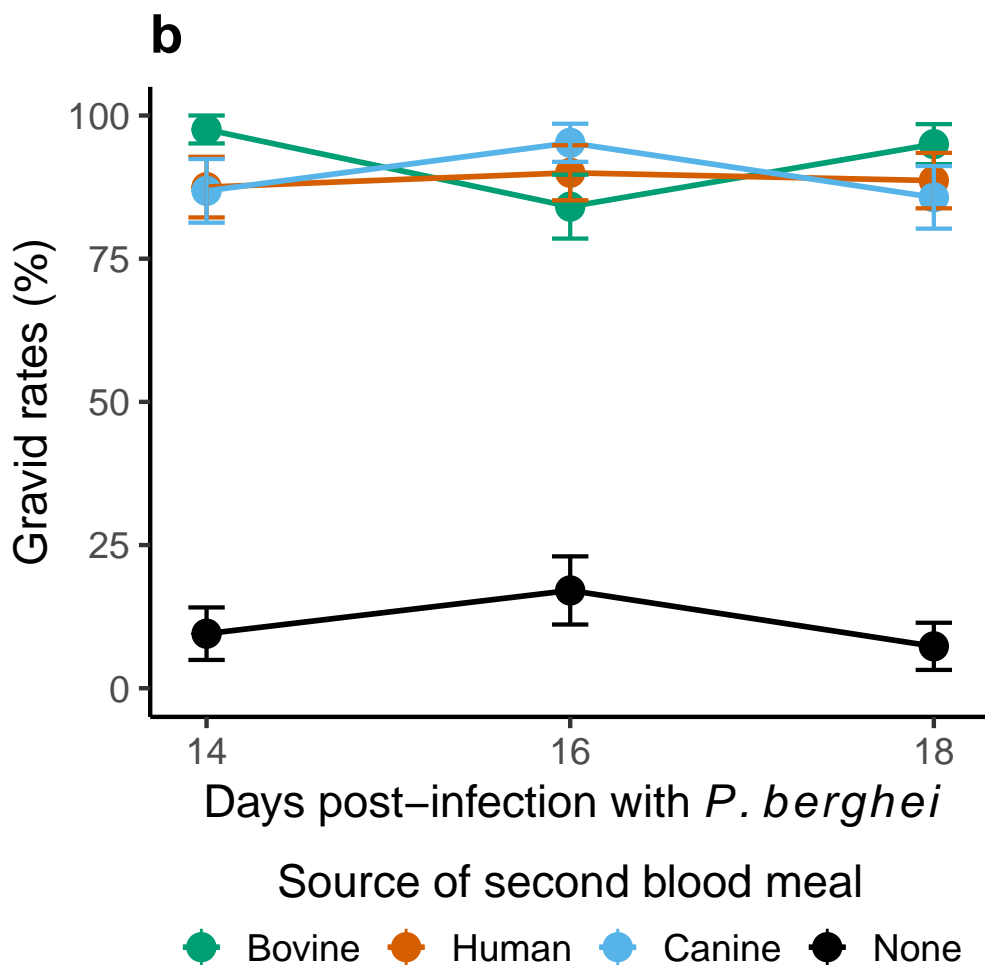

Supplement: Supplementary Fig. 2 — Feeding rates for the various blood meals (indicated by colors) were assessed as the proportion of mosquitoes with eggs in the ovaries (‘gravid’), following initial challenge with (a) P. falciparum and (b) P. berghei. Black lines depict the control group that were initially challenged with parasite, but not offered a second blood meal (‘None’); low rates of gravidity indicate high rates of oviposition prior to the second bloodmeal (for detailed rationale, refer to ‘Study design’ section under ‘Methods’). Note that gravid rates were measured from the same mosquitoes that were checked for sporozoite presence in the salivary glands (Fig. 1 and Fig. 2, also see ‘Study design’ section under ‘Methods’); as such, data represents mean and standard error from two independent replicates. See supplementary Table 1 for statistical analysis and Supplementary Table 2, Supplementary Table 3 for post hoc pairwise comparisons of the means estimated by the analyses. [file mmc2.pdf]

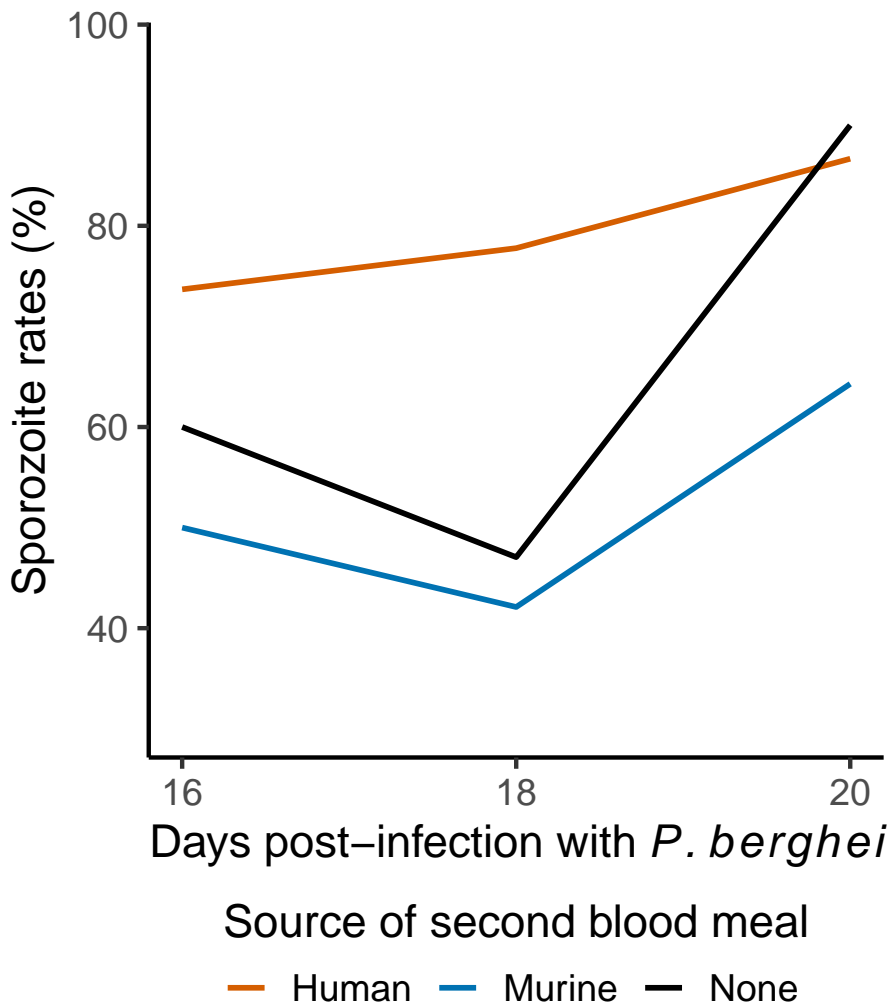

Supplement: Supplementary Fig. 3 — After the initial infectious blood meal, a second blood meal from human (red lines), canine (blue), and a mouse (dark blue) donor altered migration rates of rodent-specific P. berghei sporozoites to the salivary glands, compared to mosquitoes that were not offered a second blood meal (‘None’, black). Data is from one replicate and therefore, standard errors were not estimated. [file mmc3.pdf]
