# Supplementary material for: Blood meals from ‘dead-end’ vertebrate hosts enhance transmission potential of malaria-infected mosquitoes
Source: One Health. 2023 Jun 9;17:100582. doi: 10.1016/j.onehlt.2023.100582 (PMC10665158; doi:10.1016/j.onehlt.2023.100582)
Supplement: Supplementary Table 1 — Statistical model of feeding rates for groups challenged with either parasite, to compare proportions of mosquitoes with eggs in ovaries (‘gravid’) from total sampled (for rationale and methods to assess gravid status refer to ‘Study design’ section under ‘Materials and methods’). [file mmc4.docx]

| **Supplementary table 1** | | | | | | |
| --- | --- | --- | --- | --- | --- | --- |
|  | **Gravid rates *of P. falciparum* challenged mosquitoes** | | | **Gravid rates *of P. berghei* challenged mosquitoes** | | |
| *Predictors* | *Odds Ratios* | *CI* | *p* | *Odds Ratios* | *CI* | *p* |
| (Intercept) | 0.05 | 0.02 – 0.12 | **<0.001** | 0.13 | 0.07 – 0.22 | **<0.001** |
| Bovine blood [vs. None] | 83.72 | 43.54 – 160.98 | **<0.001** | 91.29 | 38.75 – 215.09 | **<0.001** |
| Human blood [vs. None] | 74.48 | 38.76 – 143.13 | **<0.001** | 62.01 | 28.19 – 136.42 | **<0.001** |
| Canine blood [vs. None] | 62.54 | 33.00 – 118.49 | **<0.001** | 66.42 | 29.75 – 148.28 | **<0.001** |
| Days post-infection (dpi) | 1.13 | 0.64 – 1.99 | 0.670 | 0.92 | 0.53 – 1.60 | 0.758 |
| Bovine blood * dpi | 0.59 | 0.31 – 1.13 | 0.110 | 0.94 | 0.40 – 2.23 | 0.890 |
| Human blood * dpi | 0.44 | 0.23 – 0.84 | **0.013** | 1.14 | 0.52 – 2.49 | 0.743 |
| Canine blood * dpi | 0.56 | 0.29 – 1.04 | 0.068 | 1.03 | 0.46 – 2.29 | 0.952 |
| **Random Effects** | | | | | | |
| σ^2^ | 3.29 | | | 3.29 | | |
| τ_00_ | 0.22 _replicate_ | | | 0.01 _replicate_ | | |
| Replicates | 2 | | | 2 | | |
| Observations | 1022 | | | 495 | | |
