# Supplementary material for: Blood meals from ‘dead-end’ vertebrate hosts enhance transmission potential of malaria-infected mosquitoes
Source: One Health. 2023 Jun 9;17:100582. doi: 10.1016/j.onehlt.2023.100582 (PMC10665158; doi:10.1016/j.onehlt.2023.100582)
Supplement: Supplementary Table 2 — Pairwise comparisons of gravid rates between the various P. falciparum-infected groups over time (dpi = days post-infection), based on means estimated by the model in Supplementary Table 1. [file mmc5.docx]

| **Supplementary table 2** | | | | | |
| --- | --- | --- | --- | --- | --- |
| **Comparison** | **Contrast - Reference** | **Dpi** | **estimate** | **SE** | **p.value** |
| 1 | None - Bovine | 10 | -0.8427245 | 0.0436962 | **<0.001** |
| 2 | None - Bovine | 12 | -0.8152954 | 0.0429059 | **<0.001** |
| 3 | None - Bovine | 14 | -0.7834499 | 0.0438422 | **<0.001** |
| 4 | None - Bovine | 16 | -0.7468820 | 0.0484963 | **<0.001** |
| 5 | None - Bovine | 18 | -0.7054399 | 0.0584838 | **<0.001** |
| 6 | None - Bovine | 21 | -0.6343493 | 0.0836175 | **<0.001** |
| 7 | None - Human | 10 | -0.8703899 | 0.0362690 | **<0.001** |
| 8 | None - Human | 12 | -0.8316284 | 0.0393372 | **<0.001** |
| 9 | None - Human | 14 | -0.7808002 | 0.0448906 | **<0.001** |
| 10 | None - Human | 16 | -0.7162754 | 0.0541697 | **<0.001** |
| 11 | None - Human | 18 | -0.6378487 | 0.0679994 | **<0.001** |
| 12 | None - Human | 21 | -0.4993026 | 0.0944482 | **<0.001** |
| 13 | None - Canine | 10 | -0.8199915 | 0.0481320 | **<0.001** |
| 14 | None - Canine | 12 | -0.7839430 | 0.0491017 | **<0.001** |
| 15 | None - Canine | 14 | -0.7416926 | 0.0516501 | **<0.001** |
| 16 | None - Canine | 16 | -0.6930624 | 0.0572830 | **<0.001** |
| 17 | None - Canine | 18 | -0.6382744 | 0.0671350 | **<0.001** |
| 18 | None - Canine | 21 | -0.5462504 | 0.0893233 | **<0.001** |
| 19 | Bovine - Human | 10 | -0.0276654 | 0.0421106 | 0.9131034 |
| 20 | Bovine - Human | 12 | -0.0163330 | 0.0401895 | 0.9773174 |
| 21 | Bovine - Human | 14 | 0.0026497 | 0.0375722 | 0.9998742 |
| 22 | Bovine - Human | 16 | 0.0306065 | 0.0388904 | 0.8603850 |
| 23 | Bovine - Human | 18 | 0.0675912 | 0.0507910 | 0.5434628 |
| 24 | Bovine - Human | 21 | 0.1350467 | 0.0869349 | 0.4059910 |
| 25 | Bovine - Canine | 10 | 0.0227330 | 0.0471544 | 0.9630661 |
| 26 | Bovine - Canine | 12 | 0.0313523 | 0.0434290 | 0.8883709 |
| 27 | Bovine - Canine | 14 | 0.0417573 | 0.0393154 | 0.7126953 |
| 28 | Bovine - Canine | 16 | 0.0538196 | 0.0394533 | 0.5222462 |
| 29 | Bovine - Canine | 18 | 0.0671655 | 0.0496484 | 0.5294257 |
| 30 | Bovine - Canine | 21 | 0.0880989 | 0.0829140 | 0.7124404 |
| 31 | Human - Canine | 10 | 0.0503984 | 0.0449650 | 0.6767417 |
| 32 | Human - Canine | 12 | 0.0476854 | 0.0431271 | 0.6861070 |
| 33 | Human - Canine | 14 | 0.0391076 | 0.0398671 | 0.7603632 |
| 34 | Human - Canine | 16 | 0.0232131 | 0.0398365 | 0.9372975 |
| 35 | Human - Canine | 18 | -0.0004257 | 0.0506656 | 0.9999998 |
| 36 | Human - Canine | 21 | -0.0469478 | 0.0861807 | 0.9479580 |
