# Supplementary material for: Blood meals from ‘dead-end’ vertebrate hosts enhance transmission potential of malaria-infected mosquitoes
Source: One Health. 2023 Jun 9;17:100582. doi: 10.1016/j.onehlt.2023.100582 (PMC10665158; doi:10.1016/j.onehlt.2023.100582)
Supplement: Supplementary Table 3 — Pairwise comparisons of gravid rates between the various P. berghei infected groups over time (dpi = days post-infection), based on means estimated by the model in Supplementary Table 1. [file mmc6.docx]

| **Supplementary table 3** | | | | | |
| --- | --- | --- | --- | --- | --- |
| **Comparison** | **contrast - reference** | **Dpi** | **estimate** | **SE** | **p.value** |
| 1 | None - Bovine | 14 | -0.8091793 | 0.05847327 | **<0.001** |
| 2 | None - Bovine | 16 | -0.8080066 | 0.03742776 | **<0.001** |
| 3 | None - Bovine | 18 | -0.8039053 | 0.06028329 | **<0.001** |
| 4 | None - Human | 14 | -0.7578363 | 0.06618743 | **<0.001** |
| 5 | None - Human | 16 | -0.7744462 | 0.04022415 | **<0.001** |
| 6 | None - Human | 18 | -0.7899469 | 0.0605715 | **<0.001** |
| 7 | None - Canine | 14 | -0.7771955 | 0.06397787 | **<0.001** |
| 8 | None - Canine | 16 | -0.7812983 | 0.03988145 | **<0.001** |
| 9 | None - Canine | 18 | -0.7840726 | 0.06215741 | **<0.001** |
| 10 | Bovine - Human | 14 | 0.05134306 | 0.05838495 | 0.81556261 |
| 11 | Bovine - Human | 16 | 0.03356039 | 0.03742676 | 0.80656636 |
| 12 | Bovine - Human | 18 | 0.01395843 | 0.06030173 | 0.99562756 |
| 13 | Bovine - Canine | 14 | 0.0319838 | 0.05582956 | 0.94014262 |
| 14 | Bovine - Canine | 16 | 0.02670826 | 0.03704534 | 0.88873322 |
| 15 | Bovine - Canine | 18 | 0.01983275 | 0.06189679 | 0.98861436 |
| 16 | Human - Canine | 14 | -0.0193593 | 0.06389918 | 0.99033589 |
| 17 | Human - Canine | 16 | -0.0068521 | 0.03987993 | 0.99819555 |
| 18 | Human - Canine | 18 | 0.00587432 | 0.06216987 | 0.99969756 |
