# Supplementary material for: Blood meals from ‘dead-end’ vertebrate hosts enhance transmission potential of malaria-infected mosquitoes
Source: One Health. 2023 Jun 9;17:100582. doi: 10.1016/j.onehlt.2023.100582 (PMC10665158; doi:10.1016/j.onehlt.2023.100582)
Supplement: Supplementary Table 4 — Statistical modeling of survival rates of Plasmodium-infected mosquitoes. [file mmc7.docx]

| **Supplementary table 4** | | | | | | |
| --- | --- | --- | --- | --- | --- | --- |
|  | ***P. falciparum* mortality risk** | | | ***P. berghei* mortality risk** | | |
| *Predictors* | *Risk ratio* | *95%CI* | *p* | *Risk ratio* | *95%CI* | *p* |
| Bovine blood [vs. None] | 0.96 | 0.56 – 1.65 | 0.885 | 0.98 | 0.28 – 3.38 | 0.973 |
| Human blood [vs. None] | 0.98 | 0.57 – 1.68 | 0.949 | 1.46 | 0.46 – 4.61 | 0.516 |
| Canine blood [vs. None] | 6.48 | 4.25 – 9.85 | **<0.001** | 3.47 | 1.27 – 9.49 | **0.015** |
| **Random Effects** | | | | | | |
| τ_00_ | 0.03 _replicate_ | | | 0.00 _replicate_ | | |
| Replicates | 2 | | | 2 | | |
| Observations | 1526 | | | 675 | | |
