# Supplementary material for: Blood meals from ‘dead-end’ vertebrate hosts enhance transmission potential of malaria-infected mosquitoes
Source: One Health. 2023 Jun 9;17:100582. doi: 10.1016/j.onehlt.2023.100582 (PMC10665158; doi:10.1016/j.onehlt.2023.100582)
Supplement: Supplementary Table 5 — Statistical modeling of survival rates of naïve mosquitoes (never exposed to Plasmodium) after being offered whole blood from various sources after collection in different anticoagulants (ACD = anticoagulant citrate-dextrose, EDTA = Ethylenediaminetetraacetic acid). [file mmc8.docx]

| **Supplementary table 5** | | | | | | |
| --- | --- | --- | --- | --- | --- | --- |
|  | **Risk of mortality to naïve mosquitoes under *P. falciparum* like conditions** | | | **Risk of mortality to naïve mosquitoes under *P. berghei* like conditions (20°C)** | | |
| *Predictors* | *Estimates* | *CI* | *p* | *Estimates* | *CI* | *p* |
| *Reference = not bloodfed (‘None’)* |  |  |  |  |  |  |
| Human blood in ACD | 0.16 | 0.10 – 0.26 | **<0.001** | 0.64 | 0.23 – 1.81 | 0.405 |
| Bovine blood in EDTA | 0.48 | 0.34 – 0.68 | **<0.001** |  |  |  |
| Canine blood, donor 1 in EDTA | 3.68 | 2.72 – 4.99 | **<0.001** | 4.68 | 2.22 – 9.85 | **<0.001** |
| Bovine blood in heparin | 0.42 | 0.29 – 0.61 | **<0.001** |  |  |  |
| Canine blood from donor 1 in heparin | 0.48 | 0.33 – 0.69 | **<0.001** | 1.70 | 0.73 – 3.98 | 0.221 |
| Canine blood, donor 2 in EDTA |  |  |  | 7.02 | 3.40 – 14.52 | **<0.001** |
| Canine blood from donor 2 in heparin |  |  |  | 1.83 | 0.80 – 4.18 | 0.152 |
| Replicates | 1 | | | 1 | | |
| Observations | 7050 | | | 7256 | | |
